# L2 Willingness to Communicate in the Context of Online Learning Environments: A Systematic Review

**DOI:** 10.3390/bs16020229

**Published:** 2026-02-05

**Authors:** Fang Wang, Xiaoyun Yu, Xiaoquan Pan

**Affiliations:** 1 Xingzhi College, Zhejiang Normal University, Jinhua 321004, China; violet@zjnu.cn; 2 College of Foreign Languages, Zhejiang Normal University, Jinhua 321004, China

**Keywords:** systematic review, L2 willingness to communicate, online learning environment, formal learning, informal learning

## Abstract

Given the burgeoning interest in second language willingness to communicate (L2 WTC) in the context of second language acquisition (SLA) within online learning environments, and the current lack of systematic reviews on the topic, this study employed the PRISMA framework to conduct a comprehensive review of empirical research from January 2010 to March 2025. Through an analysis of 13 examined studies, this review synthesizes key determinants of online L2 WTC into an integrated framework comprising three interrelated dimensions: intrapersonal (e.g., trait-like affective variables and cognitive variables), mediating (state-like affective, cognitive, and interactional variables), and situational (teacher supports and online learning activities). The findings notably highlight the concentrated research attention in Asian contexts and variability in measurement approaches, while underscoring the need for more experimental, idiodynamic, and longitudinal designs to better understand the dynamics of L2 WTC in digital settings. This review identifies critical methodological gaps, offering a clearer foundation for future research in technology-mediated second language acquisition.

## 1. Introduction

During the COVID-19 pandemic, an increasing number of learning activities took place in online contexts (Luan et al., 2023), thereby accelerating the integration of online learning into mainstream education. However, difficulties with online learning should not be overlooked. For example, not all students are prepared to engage in online learning; some encounter technical issues and experience feelings of isolation and reduced sociability due to the deprivation of instant face-to-face communication (Ip & To, 2025; Sitar-Tăut et al., 2024). Willingness to communicate (WTC) is widely recognized as crucial to promoting learners’ academic achievement in second language acquisition (SLA) (Peng, 2022). The last two decades have witnessed extensive discussion in the realm of WTC, propelled by significant advancements in both empirical studies and theoretical frameworks within language development studies, as well as its expanded analysis integrating linguistics, psychology, pedagogy, and other related disciplines. A growing body of bilingualism research has demonstrated that second language learning strengthens learners’ executive functions and metalinguistic awareness in the domain of cognitive development. As one of the essential objectives of SLA, second language communicative competence has received increasing attention. It refers to individuals’ ability to understand and produce both referential and social meanings of a language in accordance with its rules (Sun, 2020). It is worth noting that WTC is often the ultimate goal for second language learners (MacIntyre et al., 1998; MacIntyre & Doucette, 2010; Yang et al., 2024). Consequently, this study focuses on the potential factors influencing second language WTC (L2 WTC) in online environments. In this study, L2 WTC is defined as “a readiness to enter into discourse at a particular time, with a specific person or persons, using a specific language or mix of languages” (Henry & MacIntyre, 2024). Moreover, the online environments examined in this study include both formal online instruction where teachers and students interact synchronously via online education tools like DingTalk—a smartphone-based digital platform developed by Alibaba that integrates communication, collaborative workflows, and key pedagogical functions (e.g., live streaming, homework submission, and correction)—and informal digital settings without teacher involvement (e.g., informal digital learning of English). Notably, DingTalk was widely adopted by educational institutions during the COVID-19 pandemic due to its robust educational affordances, making it a prominent tool for online teaching (Huang et al., 2021).

**Figure 1:**
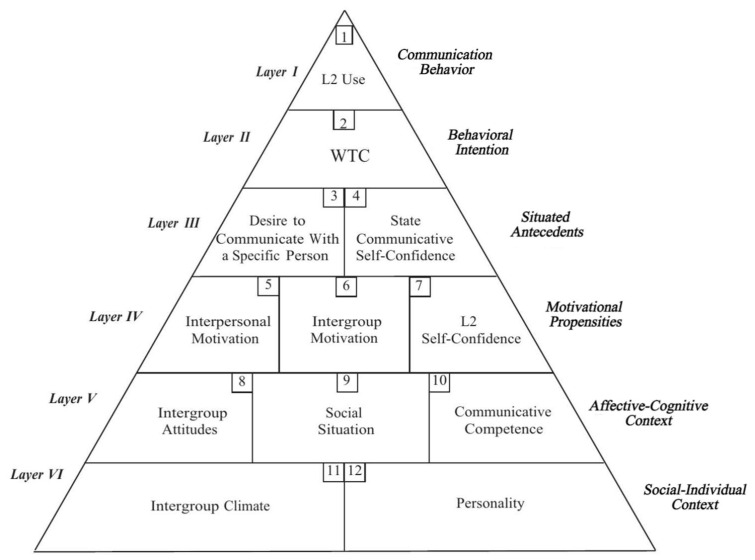
The pyramid model of L2 WTC (MacIntyre et al., 1998, p. 547).

According to the pyramid model for L2 WTC proposed by MacIntyre et al. (1998) (see Figure 1), which categorizes intrapersonal, interpersonal, and situational variables contributing to learners’ WTC into six layers, willingness to communicate is at layer two and belongs to a behavioral intention. For example, if a teacher raises a question in class, any student who raises his or her hand indicates a nonverbal communicative inclination and, therefore, should be considered as being willing to answer the question if given the opportunity. Therefore, willingness to communicate plays a pivotal role in language learning and serves as a direct predictor of learners’ actual language use behavior. WTC was originally conceived as a trait that remains stable over time, reflecting the general pattern that some people communicate frequently while others do so rarely (MacIntyre, 2020). A decade after MacIntyre’s pyramid model of L2 WTC had gained widespread adoption and acceptance, Larsen-Freeman and Cameron (2008) proposed the Complex Dynamic Systems Theory (CDSTs), which conceptualizes L2 WTC as both a dynamic and a stative construct. Building on this theory, several researchers (Yashima et al., 2018; Kruk, 2022; Taherian et al., 2023; Lee & Liu, 2024) explored contexts in which language learners are willing or unwilling to communicate. Notably, they found these two scenarios overlapped significantly, accounting for the immediate fluctuations in WTC (MacIntyre et al., 2011). In other words, students’ WTC exhibits a dynamic, context-dependent nature: the same individual may demonstrate high WTC in some situations and low WTC in others. Drawing on prior research, Henry and MacIntyre innovatively categorized WTC into two forms: trait-like WTC and state-like WTC. The trait-like WTC refers to a learner’s relatively stable, cross-situational predisposition to engage in L2 communication, reflecting a personal characteristic (e.g., a student consistently volunteers to answer questions in every English class, regardless of the topic or classmates). And the state-like WTC refers to a learner’s momentary and situation-specific willingness to communicate, which fluctuates based on contextual variables. For example, a usually quiet student feels a sudden urge to share an opinion when a highly engaging and familiar topic is raised in a small group discussion with the inspiration of the teacher.

Extensive research has indicated that higher levels of L2 WTC are positively associated with more frequent second language use in and out of the classroom, a key factor in enhancing learners’ communicative proficiency (Munezane, 2015; Yang et al., 2024). Consequently, language researchers worldwide have explored potential determinants and effective strategies to boost learners’ L2 WTC. Multiple studies have confirmed that learners’ affective variables—including foreign language enjoyment (FLE), foreign language boredom (FLB), motivation, and grit—influence L2 WTC (Lee, 2022b; Wu & Lin, 2014; M. Wang et al., 2022). With the rise of positive psychology, proposed by Seligman and Csikszentmihalyi, which regards the positive institutions, positive personality traits, and positive subjective experience as three pillars, scholars have expanded their focus to the role of learning environments, including teacher and classroom-related factors, such as teacher support, group interaction and topic selection, in improving L2 WTC (Cao, 2011; Joe et al., 2017). Conceptualized as a dynamic construct, WTC is shaped by the reciprocal interplay between individual attributes and environmental determinants (MacIntyre, 2020). Specifically, individual attributes are enduring and trait-like factors that are relatively stable across contexts, while environmental determinants are situational and transient. Based on previous research, Shirvan et al. (2019) conducted a meta-analysis to examine the moderating effects among three core variables, namely, perceived communicative competence, language anxiety, and motivation. To the best of our knowledge, most prior studies have focused on L2 WTC in offline classroom settings, utilizing cross-sectional designs to explore correlations among influencing variables. In contrast, few studies have examined L2 WTC within online formal and informal learning environments, let alone conducted a holistic systematic review.

The year 2012 marked the advent of three main massive online open course platforms—Coursera, Udacity, and edX. Their emergence stirred up a wave of enthusiasm for global online education. Additionally, the adoption of online tools, such as Duolingo, YouTube, and TikTok, has facilitated the growth of informal online learning (Meirbekov et al., 2024; Tan, 2013; Ouyang et al., 2024). Since then, online learning (both formal and informal), alongside blended learning, has become a prominent paradigm in education. Compared with traditional classroom education with a fixed schedule and curriculum, the emergence of online platforms with abundant resources has provided greater opportunities for second language learning, offering enhanced flexibility, dynamism, and accessibility (Broadbent & Poon, 2015; Lake & Dusseault, 2020; Meirbekov et al., 2024). However, concerns have been raised regarding the quality and effectiveness of online language learning, as well as learners’ self-regulation and WTC (Broadbent & Poon, 2015; Meirbekov et al., 2024; Solhi, 2024). During the COVID-19 pandemic, schools were closed temporarily to minimize face-to-face interaction. A United Nations (2020) report noted that the closure of educational institutions worldwide in April 2020 disrupted learning for 94% of the world’s students, necessitating the widespread implementation of multifaceted online learning approaches.

In view of this interest in L2 WTC and online learning environments, it is imperative to examine potential factors influencing learners’ L2 WTC in both formal and informal online learning environments. Existing studies have delved into factors related to online L2 WTC, such as online foreign language enjoyment (FLE), perceived communicative competence, online foreign language anxiety (FLA), openness to the online learning experience, teacher affective and pedagogical support, virtual and nonverbal affective supports, and learning stimuli, to name a few (Lee & Liu, 2024; Solhi, 2024; Yang et al., 2024). However, no systematic review regarding L2 WTC in online learning environments currently exists. Consequently, this study employs a systematic approach to conduct a comprehensive review of the empirical literature, aiming to identify the variables underpinning L2 WTC in online learning environments. This research seeks to advance understanding in linguistics, psychology, and education of the multidimensional nature of L2 WTC in online learning environments. Moreover, it examines theoretical frameworks and methods (including participants, measurement tools, data analysis, and results) to lay a solid foundation for future investigations in this domain.

## 2. Methods

This study adopted a systematic review approach, primarily involving qualitative content analysis of the screened articles, to collect and analyze online environment factors influencing students’ L2 WTC, covering all publications up until March 2025. Aiming for transparency in the data collection process, this study adhered to the Preferred Reporting Items for Systematic reviews and Meta-Analyses (PRISMA) framework. It systematically extracted and analyzed descriptive data, including author information, country, year of publication, underlying theories, L2 WTC measurement tools, participants, and influencing variables. The identified factors were categorized into intrapersonal, mediating, and situational dimensions: the intrapersonal dimension includes trait-like affective variables and cognitive variables; the mediating dimension comprises state-like affective variables, cognitive variables, and interactional variables; and the situational dimension encompasses teacher support and informal digital learning of English (IDLE). IDLE (Lee et al., 2024a; Lee & Chiu, 2024) refers to students’ autonomous English learning activities in extramural online environments (e.g., watching English movies in the cinema and interacting with other English users online). 

### 2.1. Search Strategy

The literature research was conducted using three reliable databases, namely, Web of Science (WOS), Scopus, and ProQuest. The search spanned from January 2010 to March 2025, focusing on L2 WTC in online learning environments. Two keywords used for searching were “second language willingness to communicate” and “online environment”, supplemented by abbreviations like “L2 willingness to communicate” and “L2 WTC”. To broaden coverage, we specified the keyword “online environment” into online formal and informal environments. Therefore, keywords like “online learning”, “online classroom”, “digital learning”, and “informal digital learning of English” were also included in the retrieval, combined with “L2 WTC”. Boolean operators (e.g., AND) were used to combine these terms, yielding a search strategy that ensured comprehensive coverage of the relevant literature while maintaining topic specificity.

### 2.2. Inclusion and Exclusion Criteria

To ensure the relevance and quality of the included articles, we adopted the following criteria. Studies were included if they concentrated on factors influencing L2 WTC in formal or informal online learning environments and were original empirical articles. Conversely, studies were excluded if: (1) full texts were unavailable or lacked the basic structural components (e.g., introduction, methodology, results, and discussion); (2) they were duplicates (removed via Zotero 7.0.32’s automated deduplication, supplemented by manual verification); (3) they were non-empirical (e.g., conference proceedings and reviews); (4) they did not specifically focus on L2 WTC, such as L3 WTC or willingness to talk/write, which fell outside this review’s scope.

### 2.3. Screening Process

Searches across the three databases yielded 191 initial records (WOS: 120; Scopus: 55; ProQuest: 16). After deduplication, 117 studies remained for further screening and were imported into Zotero and an Excel file. In the first stage, authors independently assessed record availability and type: 17 articles were excluded due to unobtainable full texts, along with 3 conference proceedings (lacking key data for analysis) and 8 reviews (secondary research, inconsistent with the focus on primary studies). In the second stage, 9 articles from non-core journals were excluded, and the remainder underwent title and abstract screening. A further 67 articles were excluded for failing to meet the inclusion criteria: 34 articles did not specifically focus on online learning environments, as these studies merely incorporated peripheral digital elements (e.g., online video playback, digital game-based activities) into traditional face-to-face classroom instruction to foster L2 WTC, which is inconsistent with the present study’s conceptualization of authentic online formal and informal learning environments as the core research context, 30 did not focus specifically on L2 WTC (e.g., general language communications or L3 WTC), and 3 articles investigated L2 WTC in digital games but in in-person classrooms and were not suitable for this topic. The most relevant and typical 13 articles were recorded by the author and validated by co-authors. A rigorous collaborative protocol guided eligibility assessment: all authors independently evaluated articles against predefined criteria, followed by cross-validation to ensure methodological consistency. Discrepancies (e.g., conflicting interpretations of measurement validity or contextual applicability) were resolved through iterative discussions until full consensus was reached, resulting in a final corpus of 13 empirical studies meeting this review’s stringent quality criteria.

### 2.4. Analysis and Coding Process

**Figure 2:**
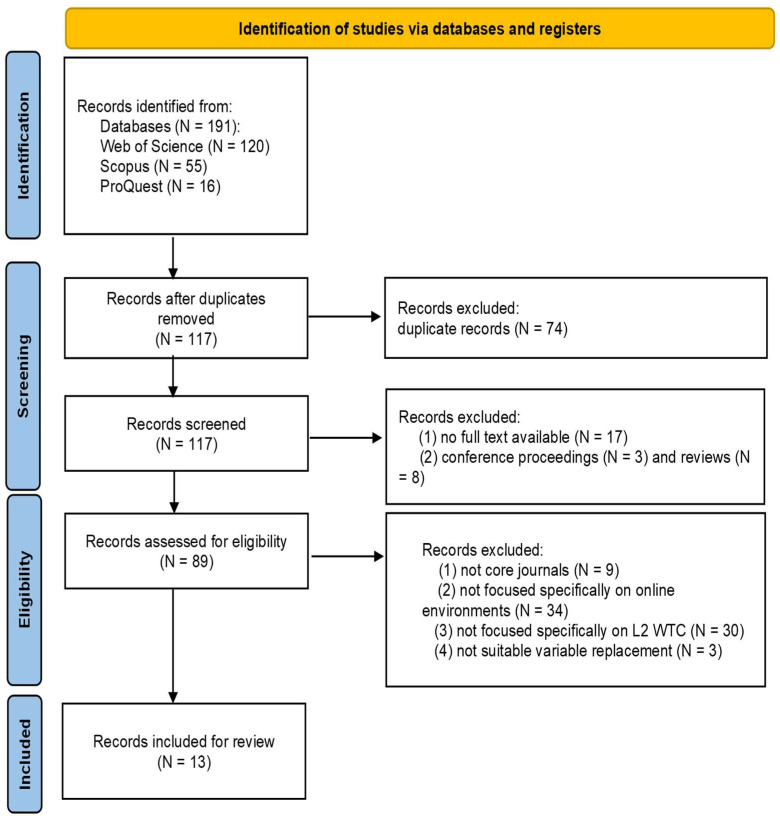
Literature search and selection process adapted from Page et al. (2021).

The 13 studies retained for final synthesis are detailed in the PRISMA flow diagram (Figure 2). An Excel worksheet was developed to extract and categorize information from the chosen articles. In the initial phase, key information was extracted, including publication year, country of origin, and participants’ educational level. Subsequently, content analysis was employed to synthesize data based on three key characteristics of each study: (1) intrapersonal, mediating, and situational variables contributing to L2 WTC in online learning environments, (2) frequently used instruments, (3) theories, measurement scales, analysis methods, and results. Finally, tables were generated to encapsulate the prevailing trends and distinctive attributes, facilitating a holistic overview of variables influencing L2 WTC in online learning environments.

## 3. Results

### 3.1. Preliminary Analyses

**Figure 3:**
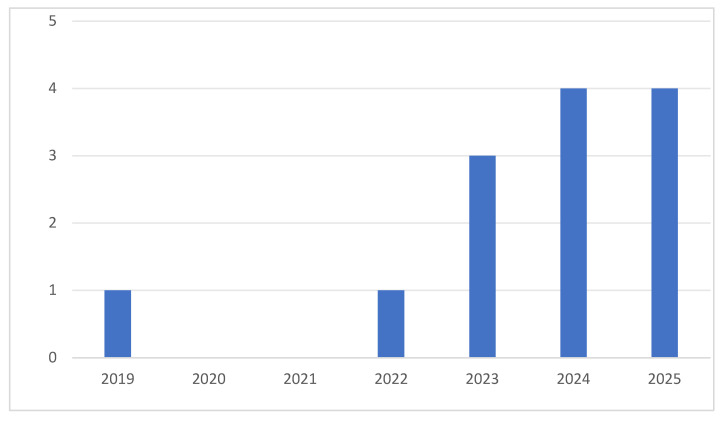
The number of analyzed publications examining online L2 WTC per year between 2019 and 2025.

The timespan of included published articles ranges from January 2019 to March 2025. The first empirical study on L2 WTC in online learning environments was released in 2019, investigating how affective variables and informal digital learning of English (IDLE) affect learners’ L2 WTC. Notably, no articles on this topic were published in 2020 or 2021, a phenomenon explainable by two key factors. First, many academic activities (e.g., international and domestic conferences, workshops, and visiting scholar programs) were cancelled due to the global pandemic, and the COVID-19 lockdowns posed significant challenges to data collection in empirical studies that rely on on-site observations, classroom audio-visual recordings, face-to-face interviews, and the administration of paper questionnaires. Second, academic research inherently involves a time lag, as both the writing and publication of a paper require substantial time investment. In the year of 2023, when people gradually went back to the workplace, focus shifted to the realm of L2 WTC in online learning environments (see Figure 3). With the widespread adoption of online language learning, scholars have moved beyond traditional offline teaching paradigms to explore the facilitative effects of online English learning activities on students’ L2 WTC. Although the number of publications on this topic is not as high as the author estimated, existing research tends to emphasize the positive impacts of online environments on L2 WTC. For example, H. Zhang and Huang (2023) examined the positive effects of online learning enjoyment and classroom interaction on L2 WTC in online classrooms, while Fu (2025) extended these effects to authentic extracurricular pragmatic contexts, promoting the normalization of L2 WTC in daily life.

**Figure 4:**
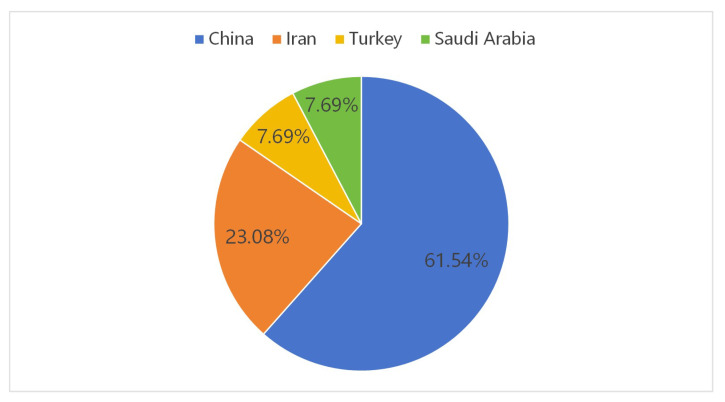
The country of origin of the examined studies.

Notably, among the 13 articles meeting the inclusion criteria, the majority (61.54%) were conducted in China, as shown in Figure 4, followed by Iran, Turkey, and Saudi Arabia. Research on online L2 WTC is cross-cultural and cross-border, with a notable concentration in emerging education markets—particularly in Asian and Middle Eastern countries. This suggests a perceived urgency in these regions to develop second language proficiency for international communication. A closer inspection revealed all Chinese publications involved collaborations with Ju Seong Lee, whose research focused on Computer-Assisted Language Learning (CALL), informal digital learning of English (IDLE), and English as an international language. This indicates that Professor Lee is an authoritative figure with considerable experience in this field, but also reveals the pitfall of a lack of diversity in the academic ecosystem.

**Figure 5:**
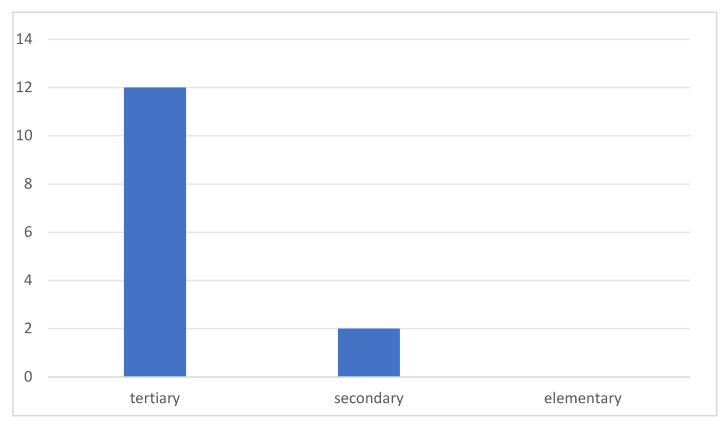
Educational levels of participants.

Regarding participants’ educational backgrounds, nearly all studies included tertiary learners, including undergraduate and postgraduate students, ranging from 18 to 39 years old (see Figure 5). Two studies (Lee et al., 2024a, 2024b) investigated variables at the secondary level, ranging from 12 to 17 years old, with Lee et al. (2024a) covering both tertiary and secondary participants, but neglected the elementary level. This indicates a facilitation bias in academic research, which much of the extant research concentrates on easily accessible participants, thus neglecting the distinct requirements of elementary education.

### 3.2. Summary of 13 Studies

As is clearly presented in Table 1, variables from the 13 examined studies were categorized into intrapersonal dimensions, mediating dimensions, and situational dimensions. This categorization is based on Bronfenbrenner’s (1993) ecological systems theory, which posits that individual psychological development is profoundly influenced by the dynamic social environment. This categorization serves as an analytical heuristic: a conceptual tool designed to systematically organize and synthesize diverse variables for clearer analysis, rather than a rigid, mutually exclusive classification. Specifically, intrapersonal dimensions refer to relatively stable traits and immediate psychological states inherent to language learners, including affective variables (e.g., individuals’ foreign language enjoyment, anxiety, and grit) and cognitive variables (e.g., openness to the online learning experience and basic psychological needs). Mediating dimensions refer to variables that connect the external environment and learners’ L2 WTC, including state-like affective variables (e.g., FLE, FLA, and FLB), cognitive variables (e.g., demotivation, confidence, and self-efficacy beliefs), and interactional variables (e.g., teacher–student interactions and student–student/group interactions). Situational dimensions refer to external conditions acting on language learners in specific learning contexts, in particular online learning environments, including teacher support variables (e.g., affective support and pedagogical support) and online activities, such as IDLE (focusing on language learning beyond classrooms).

The heuristic’s flexibility is evident in boundary cases where variable classification depends on context: for example, FLE may function as an intrapersonal trait (e.g., a learner consistently experiences enjoyment in English learning) or a mediating state (e.g., enjoyment triggered by an engaging online task, linking teacher support to L2 WTC). Similarly, self-efficacy beliefs can be an intrapersonal cognitive trait (stable belief in one’s L2 abilities) or a mediating variable (strengthened by successful IDLE engagement to enhance L2 WTC). A key finding is the multifaceted role of affective variables: as stable traits, they form an emotional baseline moderating environmental effects; as situational states, they mediate links between external interventions (e.g., teacher support) and L2 WTC. This confirms the core hub position of affect in the interaction between individuals and the environment emphasized by positive psychology theory (Seligman & Csikszentmihalyi, 2000).

Based on the examined 13 studies, two analytical pathways for investigating online L2 WTC emerged. The first path is the direct effect pathway, investigating the correlation between specific variables and L2 WTC. This pathway focuses on relatively stable and enduring dispositional variables within learners or situational variables, regarding them as antecedent predictive variables or foundational conditions influencing L2 WTC. The core of the research lies in exploring how the independent variable directly shapes or constrains their communicative tendencies in online contexts. The examined studies (Fattahi et al., 2023; Lee & Hsieh, 2019), serve as typical cases in this regard. The first study investigated the correlations among trait-like FLE, trait-like FLB, and online L2 WTC. The second study not only discussed the correlation between affective variables and L2 WTC, but also compared learners’ L2 WTC in different learning contexts.

The other path is the indirect effect pathway based on the interaction between environments and individuals. This pathway adopts a more dynamic and interactive perspective, examining how situational variables enhance L2 WTC by triggering or altering learners’ internal malleable and variable mediating states. The core theoretical value of this pathway lies in revealing that L2 WTC is not solely determined by individuals’ inherent traits, but rather a dynamically constructed process through the real-time mediation of internal states in specific contexts. The following examined studies are typical examples: (H. Zhang & Huang, 2023; Lee et al., 2024a, 2024b). The first study employed state-like FLE and group interaction as mediators, investigating their functions between perceived teachers’ enthusiasm and L2 WTC. They also examined the moderation of gender, weekly self-online learning time, and learning achievement, providing a certain paradigm for future research. The second study also examined learners across gender groups, with its primary aim being to compare how affective variables mediate between IDLE and L2 WTC across genders and educational levels. The third study employed a similar mediation model but focused on specific sub-dimensions of FLE to investigate its underlying mechanism.

Additionally, some scholars (Bensalem et al., 2025; Noughabi & Ghasemi, 2025; Solhi, 2024; Zadorozhnyy & Lee, 2025) integrated both pathways, investigating direct correlations and mediated indirect effects. Most studies (Bensalem et al., 2025; Fattahi et al., 2023; Fu, 2025; Lee et al., 2024b; Lee & Hsieh, 2019; Solhi, 2024; H. Zhang & Huang, 2023) adopted an emotional perspective (influenced by Positive Psychology theory), with FLE as the most frequently cited variable (often a direct/full mediator). Drawing on the control–value theory and broaden-and-build theory, some studies (Bensalem et al., 2025; Lee et al., 2024a; Solhi, 2024) incorporated negative emotions when analyzing, holding the conviction that negative emotions like boredom and anxiety would diminish L2 WTC to a certain degree. A thorough investigation was conducted regarding the themes leading to the prevalence of negative emotions; it revealed a multitude of variables, including lack of interaction, dull atmosphere, internet connection issues, uninteresting topics, and so forth. Collectively, these variables have been identified as detrimental to L2 WTC (Fattahi et al., 2023). However, it is argued that language learners experience a lower degree of negative emotions due to the online supplementary resources accessible and the online creation of a low affective-filter environment, compared with offline.

Two studies investigated the fluctuations of L2 WTC with an idiodynamic and longitudinal design employed, respectively, based on the Complex Dynamic Systems Theory (CDSTs), thus compensating for the limitations of traditional cross-sectional quantitative research (Lee & Liu, 2024; Taherian et al., 2023). Given that the learning process of SLA is naturally dynamic, it is logical that the behavioral intention of willingness to communicate is a highly fluid and developmental process as well. Therefore, it is imperative to measure the trace and fluctuations of variables contributing to L2 WTC, paving the way for the implementation of conducive pedagogical interventions. Information about frequently used scales for measuring L2 WTC is presented in Table 2 below.

### 3.3. Theories, Participants, Tools, Data Analysis, and Results of 13 Studies

As illustrated in Table A1 (see Appendix A), the 13 studies included 5762 participants, with sample sizes ranging from 7 (idiodynamic study) to 1265 (a large-scale secondary/tertiary study). Most studies had more female participants and focused on monolingual learners, while Zadorozhnyy and Lee (2025) examined bilinguals. Participants included university undergraduates and postgraduates as well as secondary school learners. A majority of the included articles adopted a cross-sectional research design, employing questionnaire-based surveys, while one study utilized an idiodynamic method (Lee & Liu, 2024) and another applied a longitudinal approach (Taherian et al., 2023), both from a dynamic perspective. Following data collection, researchers conducted validity assessments of questionnaires, retaining valid instruments for subsequent analysis. Descriptive statistics were first employed to characterize sample demographics, followed by Pearson correlation analysis to examine variable relationships. For construct validation, confirmatory factor analysis (CFA) was utilized in 53.85% of cases (n = 7), while exploratory factor analysis (EFA) was applied in 15.38% (n = 2) to explore latent structures. The remaining 30.77% (n = 4) either adopted validated scales or used self-reported measures. Structural equation modeling (SEM) was the dominant analytical approach, appearing in 61.54% (n = 8) of the studies due to its capacity to test complex theoretical frameworks.

## 4. Discussion

This systematic review aims to synthesize and critically evaluate existing empirical research on L2 WTC and attempts to summarize the current independent variables in online learning environments via a conceptual analytical heuristic—intrapersonal, mediating, and situational dimensions—grounded in Bronfenbrenner’s Ecological Systems Theory. As a heuristic, this framework is not intended to impose fixed categorization but to provide a structured lens for synthesizing diverse variables, highlighting their dynamic interactions while acknowledging boundary cases where classification depends on contextual factors. For example, sometimes learners’ affective variables are stable and belong to personal traits. If L2 learners with low academic achievement in foreign language learning are not familiar with online learning environments or the interfaces of learning applications, they may experience relatively higher levels of anxiety and boredom that persist over an extended period. In contrast, affective variables appear to be state-mediating variables at times. For instance, learners feel less anxious if they are told that the specific online task is simply a practice rather than a test. A wide range of situational variables arises during the learning process and interaction with the environment, thus indicating the pivotal role of the teacher as a facilitator. For example, enthusiastic teachers are capable of constructing a conducive and supportive learning atmosphere, fostering students’ engagement and academic performance in class. The form of teachers’ corrective feedback has been found to affect students’ emotions to a certain degree, which might raise positive or negative emotions, contributing to learners’ willingness or unwillingness to communicate in class regardless of their language proficiency (Zare et al., 2022).

Foreign language enjoyment (FLE) refers to the extent to which the learner feels a sense of enjoyment, satisfaction, and involvement in the process of language learning (S. Li et al., 2022). According to the broaden-and-build theory proposed by Fredrickson (2001), positive emotional states expand cognitive–behavioral repertoires and enhance learners’ attentional breadth, thereby fostering proactive behaviors like initiating questions with the target language, whereas negative emotions exert constrictive effects on psychological flexibility. Furthermore, FLE can also alleviate a sense of anxiety, boredom, and demotivation that have been confirmed to influence various aspects of language learning and classroom dynamics (Y. Wang et al., 2021), such as motivation, resilience, student engagement, academic achievement, and L2 WTC. Except for the above benefits from overall FLE, Botes et al. (2021) classified FLE into three sub-dimensions based on the degree of psychological needs: (1) teacher appreciation, defined as the extent to which L2 learners believe their English teachers are meeting their psychological needs; (2) personal enjoyment, referring to the degree to which L2 learners derive pleasure from English learning; and (3) social enjoyment, denoting the extent to which L2 learners’ social psychological needs are satisfied in the English classroom. Based on this classification, Lee et al. (2024b) found that all three categories of enjoyment partially mediate the relationship between receptive and productive IDLE and L2 WTC across not only non-digital (e.g., in-class and out-of-class settings) but also digital (e.g., social media platforms) contexts, with the latter having emerged as prevalent communicative arenas for contemporary language learners. Notably, FLE is most frequently investigated as a state-like mediating variable in online environments, rather than a stable intrapersonal trait.

Foreign language boredom (FLB) and anxiety (FLA) are both negative emotions, undermining learners’ L2 WTC. Currently, FLB is considered to be the most intense and common affective variable in SLA (Fattahi et al., 2023), and bored learners are characterized by feeling uninvolved, distracted, and unwilling to engage in teacher-proposed activities, thus avoiding teacher–student interaction and group interaction. Similarly, FLA is also a prominent and prevalent indicator, appearing at each learner proficiency level. It is naturally a transient and situational variable that might be influenced by the teacher’s feedback, the speaking atmosphere, and the familiarity of the topic. Solhi (2024) found that anxiety is at the mercy of a combination of internal factors like self-perceived competence and achievement, and external factors, such as classroom interaction. Furthermore, learners may demonstrate elevated levels of FLA in online environments, owing to their potential experience of diminished perceptions of relatedness and autonomy in the absence of in-person interactions with L2 instructors and peers. With reference to control–value theory, enjoyment, boredom, and anxiety are multidimensional emotions rather than merely reflecting positive or negative. That is to say, FLE embodies positive activating process-oriented achievement affect, whereas FLB constitutes negative deactivating process-related emotional states, and FLA represents negative activating outcome-oriented achievement emotions (C. Li & Wei, 2023).

Grit is defined as the perseverance and resilience in confronting adversities alongside sustained commitment to long-term objectives (A. L. Duckworth & Quinn, 2009). Empirical evidence indicates that grit demonstrates no significant correlations with gender, academic standing, and personal intelligence but has something to do with age (Teimouri et al., 2022). Gritty students experience much happiness and curiosity, and score higher in self-esteem. It has been found that grit is strongly related to positive emotions and resilience, combined with a weak but positive correlation with academic performance and success in SLA. Gritty people are more persistent in the long-term goal and embark on a higher educational aspiration, triggered by internal motivation, and thus are less likely to give up. Bensalem et al.’s (2025) study suggested that grit is responsible for L2 WTC in online environments.

In terms of cognitive variables, openness to online learning environments represents students’ open-mindedness to embrace new environments with the rapid growth of digital development. Given that openness to experiences is conceptualized as a dispositional tendency to embrace novel approaches and engage with unfamiliar experiences (McAdams, 2015), individuals characterized by this trait are inherently inclined to interact with their surrounding environments in a curious and intrinsically motivated manner. When extended to the context of online learning, such dispositional openness is particularly salient: it fosters a heightened willingness to explore the novel instructional paradigms and technological configurations inherent in online educational settings. Consequently, openness to online experiences serves a pivotal role in mitigating the potential challenges associated with transitioning from traditional face-to-face learning to online modalities. People who are more open to new experiences are more likely to be attracted to social networking sites (Correa et al., 2010), accelerating the probability of being in contact with IDLE activities like English movies and posts, providing authentic and low-anxiety second language interaction opportunities, subsequently fostering L2 WTC by exposing learners to real-world language use and reducing apprehension toward L2 interaction. Additionally, openness to the online learning experience elevated learners’ self-efficacy and enhanced online engagement (Audet et al., 2021), which creates a positive cycle wherein openness to the online learning experience drives IDLE activities, elevates self-efficacy, and ultimately amplifies both L2 WTC and sustained learning engagement.

As illustrated by self-determination theory (SDT), basic psychological needs (BPNs) are “innate psychological nutrients critical for healthy mental growth” that promote psychological and academic success when fulfilled. Specifically, the need for autonomy, competence, and relatedness operate synergistically to shape L2 WTC in online environments through both direct and indirect pathways, as supported by recent empirical evidence (Fu, 2025) and consistent with the theoretical and empirical underpinnings of previous studies conducted by Shirvan and Alamer (2024). Autonomous selection of learning tasks and control over communication rhythms in online settings reduce external pressure and strengthen learners’ sense of agency. Online positive feedback, incremental learning achievements, and acquisition of communicative skills alleviate foreign language anxiety and enhance confidence in effective online interactions. Inclusive online learning communities foster learners’ sense of value and interpersonal connection, mitigating fear of judgment and constructing a psychologically safe communicative environment. Furthermore, as highlighted by Fu (2025), the positive effects of BPNs on L2 WTC are mediated and amplified by affective variables, such as foreign language enjoyment and grit, enabling learners to maintain resilience amid online communicative challenges and further deepen their willingness to actively engage in L2 interactions.

Demotivation and L2 confidence serve as significant cognitive variables that shape EFL learners’ online L2 WTC. Demotivation, characterized as a loss of previously held interest and effort in L2 learning (Dörnyei & Ushioda, 2021), can directly undermine learners’ readiness to engage in online communication by diminishing their intrinsic drive and perceived value of L2 use (Solhi, 2024). In Solhi’s (2024) study, L2 demotivation emerged as a strong predictor of reduced L2 WTC in online contexts, suggesting that demotivated learners are less inclined to initiate or sustain interactions in virtual classrooms. This aligns with earlier observations that demotivating factors, such as uninteresting materials or inadequate teacher support, can lower learners’ sense of autonomy and relatedness, thereby impairing their L2 WTC (Dörnyei, 1998; X. Zhang et al., 2020). Conversely, L2 confidence, which was defined as learners’ beliefs in their ability to communicate effectively in the L2 (MacIntyre et al., 1998), plays a facilitative role in fostering online L2 WTC. Lee and Hsieh (2019) found that L2 self-confidence significantly predicted L2 WTC in digital settings, indicating that confident learners are more willing to participate in online exchanges even in the absence of physical cues. This confidence may mitigate the anxiety often associated with online speaking contexts, enabling learners to perceive digital environments as less threatening and more conducive to communication (Lee & Hsieh, 2019; Reinders & Wattana, 2014). Thus, while demotivation acts as a barrier by eroding learners’ motivational and emotional resources, L2 confidence serves as an enabling variable that enhances their perceived competence and willingness to engage in online L2 interaction.

Self-efficacy beliefs refer to the degree of belief in one’s ability to succeed in a given situation (Bandura, 1997), functioning as a pivotal cognitive variable influencing L2 learners’ online L2 WTC. Learners’ past success, vicarious experiences, positive feedback, and emotional and physiological states are interwoven in the contribution to self-efficacy. In online L2 environments, self-efficacy beliefs enhance L2 WTC by fostering a sense of confidence and reducing foreign language anxiety, thereby encouraging learners to initiate and sustain interactions (Lee & Hsieh, 2019). Specifically, when learners engage in informal digital learning activities (e.g., watching English videos or participating in online discussions), their past successful experiences reinforce their beliefs in their L2 abilities, which in turn promotes L2 WTC (Solhi, 2024). Moreover, self-efficacy often operates indirectly by mediating the impact of external environmental variables, such as teacher support or digital affordances, on L2 WTC. For instance, supportive online contexts that provide positive feedback and manageable challenges can strengthen learners’ self-efficacy beliefs, which subsequently elevate their willingness to communicate (X. Zhang et al., 2020). Thus, self-efficacy beliefs not only directly motivate online L2 WTC but also serve as a psychological mechanism through which supportive digital environments enhance learners’ communicative engagement.

Building upon ecological systems theory (Bronfenbrenner, 1979), interactional variables (teacher–student interactions and group interactions) serve as a critical mediating dimension through which external situational variables shape L2 WTC in online learning environments. A harmonious teacher–student interaction is conducive to the psychological well-being of both the teacher and students, thereby increasing the teacher’s enthusiasm, which inspires learners’ engagement and L2 WTC (H. Zhang & Huang, 2023), subsequently contributing to positive learning outcomes. For example, an enthusiastic teacher is inclined to provide constructive and productive feedback, employs a variety of teaching strategies, and is concerned about learners’ progress and achievements. Conversely, group interactions, often realized through peer collaboration, group discussions, or informal digital exchanges, provide low-stakes opportunities for language practice and mutual support, reinforcing communicative self-efficacy and sustained engagement (Lee et al., 2022). Crucially, these interactional processes mediate the influence of external situational variables on learners’ L2 WTC. For example, a teacher’s instructional and emotional support can strengthen the quality of teacher–student interaction, which in turn promotes a classroom climate conducive to risk-taking and voluntary communication (Reinders & Wattana, 2014). Thus, interactional variables function not merely as communicative episodes, but also as relational mechanisms that translate environmental resources into internalized psychological readiness for L2 use in online environments.

The external situational dimensions include teacher support and online English learning activities. Teacher support, integrating both emotional support (e.g., encouragement, empathy) and pedagogical support (e.g., clear instruction, resource provision), establishes a secure base for learners. This support directly influences the learning atmosphere and, as discussed, works significantly through the mediation of positive teacher–student interactions to bolster learners’ sense of belonging and reduce inhibitory anxiety. Separately, the nature and design of online learning activities—such as interactive digital games, IDLE tasks, or structured cross-cultural exchange projects—directly shape the affordances of the learning environments. These activities are beneficial in creating a low affective-filter environment, dispelling learners’ negative emotions, thereby augmenting L2 WTC in online classrooms (Reinders & Wattana, 2014). Furthermore, Lee et al. (2022) conducted a cross-cultural comparative study of L2 WTC and reported that the Taiwanese are more willing to communicate in digital settings, compared with the Koreans, suggesting the influence of teaching instruction mode and cultural values in SLA, which was echoed by Hsu’s (2007) study about differences in WTC between American and Taiwanese university students. Learners are more willing to communicate and initiate cross-cultural communication if they embrace an explicit manner and perceive the teacher’s support and encouragement. Conversely, an accuracy-oriented teaching pedagogy and exam-oriented teaching approach can exaggerate learners’ anxiety, subsequently reducing students’ L2 WTC in online classes. In conclusion, teacher support establishes emotional and pedagogical foundations by reducing foreign language anxiety, enhancing belonging, and guiding IDLE integration, while IDLE activities lower the affective filter, reduce FLE and FLB, and provide engaging communication opportunities. Their synergistic interaction, guided by broader pedagogical values, creates a learning ecosystem that fosters sustained L2 WTC development. These findings highlight the need for online L2 instruction to prioritize both high-quality teacher support and the strategic integration of IDLE activities, thereby leveraging the external situational dimension to optimize learners’ communicative potential.

### 4.1. Implications

This systematic literature review provides valuable insights for shaping future research agendas on L2 WTC in online learning environments. As conceptualized in the pyramid model of L2 WTC and Complex Dynamic Systems Theory (CDSTs), learners’ overall L2 WTC can be understood as a combination of trait-like WTC and state-like WTC, affected by a series of underlying internal and external variables. This review affirms and extends MacIntyre et al.’s (1998) pyramid model by empirically validating a dynamic, multi-layered variable ecosystem influencing L2 WTC in online environments. The proposed classification—distinguishing relatively stable intrapersonal dimensions, dynamic mediating dimensions, and external situational dimensions—provides a refined heuristic model. Crucially, this categorization challenges static variable attribution, highlighting that affective variables can function as either trait-like dispositions or state-like mediators, depending on contextual triggers (e.g., task design, teacher support). Educators should consider the function of each variable and boost learners’ foreign language enjoyment, motivation, confidence, and self-efficacy so as to enhance their state-like L2 WTC, eventually improving their trait-like L2 WTC in the long run. Future theorizing should also embrace the fluidity of categorization, investigating how variable roles shift across different online pedagogical tasks and temporal scales.

The complexity of variable interactions necessitates methodological pluralism in future research. To capture dynamics within and across dimensions, future studies should combine longitudinal designs with high-density data collection (e.g., experience sampling, stimulated recall) to track how situational variables trigger mediating state fluctuations and subsequent impacts on state-like L2 WTC. Furthermore, researchers should employ advanced analytical techniques, such as latent growth modeling or dynamic systems analysis, to better disentangle mediation and moderation effects among the identified variables.

To enhance learners’ L2 WTC in online environments, educators should design activities to enhance foreign language enjoyment by integrating autonomy-supportive tasks and optimally challenging content. Sustained project-based learning is recommended to cultivate grit, with obstacles framed as integral to skill development and persistence celebrated alongside outcomes. To promote openness to the online learning environment and meet basic psychological needs, pre-course and in-course orientation activities on digital tools and pedagogies are essential to reduce novelty anxiety.

Additionally, educators need to construct low-affective-filter environments using formative, non-evaluative practice tasks to alleviate foreign language anxiety, while combating foreign language boredom through diversified activity formats. Real-time support and process-focused feedback are critical for emotional regulation. Early low-stakes communication tasks, paired with clear models and scaffolds, help build situational confidence. Teachers should also provide multi-faceted support, integrating technical, affective, and pedagogical support, with visible enthusiasm to enhance classroom climate and L2 WTC. Furthermore, informal digital learning of English (IDLE) should be strategically integrated: assign theme-aligned IDLE activities via Duolingo and TED talks, and establish shared online learning forums and English learning communities to encourage sharing and thereby transforming solitary learning into socially mediated practice. A communication-oriented pedagogy is imperative, shifting from accuracy-dominated, exam-centric instruction to prioritizing meaningful tasks with authentic purposes (e.g., problem-solving). This approach values participation, reduces foreign language anxiety, and aligns situational demands with fostering L2 WTC.

### 4.2. Limitations

Although this study represents the first integrative attempt to synthesize L2 WTC research in the context of online environments, potential gaps exist due to constraints in the availability and accessibility of the relevant literature. First and foremost, the scope of the reviewed literature is confined by specific inclusion criteria and the restriction to English-language publications. Consequently, the exclusion of non-English research may have resulted in the omission of potentially valuable insights. Nevertheless, every effort was made to incorporate all relevant studies within the defined scope of this research. Second, the reviewed studies exhibit an imbalance in participant demographics. Although this review managed to cover a broad range of student populations, no studies focusing on elementary-level learners were identified, with a predominant focus on tertiary-level learners. Third, while the systematic review process adhered to PRISMA guidelines, the final sample of 13 studies, though rigorously selected, is relatively small for quantitative synthesis and may be susceptible to publication bias. To address this, we conducted the content analysis, systematically comparing how each study examined variables influencing L2 WTC in online environments, summarized their similarities and differences, and categorized their approaches into two distinct analytical pathways. Future inquiries might consider supplementary methods, such as bibliometric visualization—a methodological approach that employs spatial and network representations to map and analyze scholarly literature patterns via tools like VOSviewer 1.6.20 and CiteSpace 6.3.1—to explore publication trends and thematic clusters within this domain.

## 5. Conclusions

This study reviewed 13 empirical studies on factors influencing L2 WTC in online environments, linking corresponding cited theories, participants, measurement tools, and analysis methods. We have found that intrapersonal dimensions, including affective variables (foreign language enjoyment, anxiety, and grit) and cognitive variables (basic psychological needs and openness to online learning environments), are relatively stable and could influence L2 WTC directly. Mediating dimensions are state-like variables, including affective variables (foreign language enjoyment, anxiety, and boredom), cognitive variables (demotivation, L2 confidence, and self-efficacy beliefs), and interactional variables (teacher–student interactions and group interactions). Additionally, situational dimensions are contextual and exerted by the external environment, including teacher support (affective support and pedagogical support) and online learning activities, such as informal digital learning of English (IDLE). The mediating dimensions and situational dimensions are usually intertwined and work together to enhance learners’ L2 WTC in online learning environments. MacIntyre et al.’s (1998) pyramid model for WTC and the Complex Dynamic Systems Theory (CDSTs) are the most cited for analysis within this topic. Since the research on this topic in online environments is relatively scarce and scattered across different countries, most measurement tools for assessing L2 WTC are adapted from different scholars. Furthermore, while all scholars used quantitative methods, most of their studies merely adopted a cross-sectional study design; only two articles evaluated the fluctuations and underlying variables contributing to L2 WTC over time with a longitudinal design. This study provides valuable insights for improving students’ L2 WTC within the online environment, offering practical implications for educators to create an engaging and interactive classroom environment.

## Figures and Tables

**Table 1:** Categorization of variables influencing L2 WTC in each examined study.

No	(Authors, Year), Country	Intrapersonal Dimensions	Mediating Dimensions	Situational Dimensions
1	(H. Zhang & Huang, 2023), China		online foreign language enjoyment and perceived group interaction	perceived teacher’s enthusiasm
2	(Fattahi et al., 2023), Iran	foreign language enjoyment		
3	(Solhi, 2024), Turkey	foreign language anxiety	L2 demotivation	
4	(Lee & Hsieh, 2019), China	grit	L2 self-confidence and L2 anxiety	
5	(Bensalem et al., 2025), Saudi Arabia	grit	foreign language enjoyment and boredom	
6	(Lee & Liu, 2024), China	openness to the online learning experience and interpersonal and intrapersonal factors		teacher affective and pedagogical support and virtual and nonverbal affective supports and learning stimuli
7	(Noughabi & Ghasemi, 2025), Iran		grit	informal digital learning of English
8	(Lee et al., 2024b), China		foreign language enjoyment (teacher appreciation, personal enjoyment, and social enjoyment)	informal digital learning of English
9	(Zadorozhnyy & Lee, 2025), China		self-efficacy beliefs	informal digital learning of English
10	(Lee et al., 2022), China			English environment and teaching practice
11	(Fu, 2025), China	basic psychology needs and grit	enjoyment	informal digital learning of English
12	(Lee et al., 2024a), China		foreign language enjoyment and foreign language anxiety	informal digital learning of English
13	(Taherian et al., 2023), Iran		foreign language boredom	informal digital learning of English

**Table 2:** Frequently used instruments.

Dimension of Measurement	Name of Instruments (Authors, Year)	Research Examples
L2 WTC inside classrooms	L2 willingness to communicate scale in online Chinese class (Khajavy et al., 2018; McCroskey, 1992), n = 1	Perceived teachers’ enthusiasm and willingness to communicate in the online class: the mediating role of learning enjoyment and group interaction for Chinese as a second language
	L2 willingness to communicate scale (Peng & Woodrow, 2010), n = 1	Nothing ventured, nothing gained: the impact of enjoyment and boredom on willingness to communicate in online foreign language classrooms
	L2 WTC scale modified for online classes (Lee & Hsieh, 2019), n = 2	Informal digital learning of English and L2 willingness to communicate: the roles of emotions, gender, and educational stage
	L2 WTC scale (Y. Wang et al., 2021; Lee & Drajati, 2019a), n = 2	Modeling the contributions of grit, enjoyment, and boredom to predict English as a foreign language students’ willingness to communicate in a blended learning environment
	L2 WTC scale (Lee & Drajati, 2019b), n = 1	Enjoyment and grit as mediators between informal digital learning English, basic psychology needs, and willingness to communicate among English as a foreign language university students
	L2 WTC questionnaire (Ryan, 2009; Yashima, 2009; Peng, 2013; Lee & Lee, 2020), n = 3	Understanding willingness to communicate in L2 between Korean and Taiwanese students
	Willingness to communicate questionnaire (MacIntyre et al., 2001), n = 1	Informal digital learning of English and EFL learners’ willingness to communicate: investigating the mediating role of L2 grit
	L2 WTC scale (Lee, 2022b), n = 2	Foreign language enjoyment as a mediator between informal digital learning of English and willingness to communicate

## Data Availability

The original contributions presented in this study are included in the article. Further inquiries can be directed to the corresponding authors.

## Appendix A

**Table A1:** Theories, participants, tools, analysis methods, and results.

No.	Sources	Theories	Participants	Tools	Data Analysis	Results
1	Perceived teachers’ enthusiasm and willingness to communicate in the online class: The mediating role of learning enjoyment and group interaction for Chinese as a second language	The pyramid model of WTC	473 Chinese major university students from Vietnam and Thailand (males = 119; females = 354).	Scale of WTC in the online Chinese class from previous studies (Khajavy et al., 2018; McCroskey, 1992); Scale of perceived group interaction (Brawley et al., 1987; Den Brok et al., 2006); Scale of learning enjoyment (C. Li et al., 2018); Scale of perceived teachers’ enthusiasm (Deci & Ryan, 1985; Kunter et al., 2008).	SEM; CFA multiple group analysis.	Learning enjoyment and perceived group interaction fully mediate the relationship between perceived teachers’ enthusiasm and WTC, and perceived group interaction shows a stronger mediating effect. Gender, weekly self-online learning time, and learning achievement significantly moderate the relationship between perceived teachers’ enthusiasm, learning enjoyment, perceived group interaction, and WTC.
2	Nothing ventured, nothing gained: The impact of enjoyment and boredom on willingness to communicate in online foreign language classrooms	MacIntyre et al.’s (1998) model of WTC; control–value theory of achievement; self-determination theory (SDT)	469 university students with 61% female participants; upper-intermediate proficiency in English (n = 248), followed by intermediate (n = 207), expert (n = 12), and elementary proficiency (n = 2); 20 students took the follow-up semi-structured interview.	L2 WTC scale (Peng & Woodrow, 2010); boredom in practical English language classes (Pawlak et al., 2020); foreign language enjoyment scale (Dewaele & MacIntyre, 2014).	Multiple linear regression; dominance analysis (examines which predictor variable is relatively most important; mediation analysis.	FLB had a considerably stronger effect on L2 WTC. The strong quantitative effect of FLB was to some extent mitigated by FLE but FLB seems to be severely detrimental to the willingness to communicate of online L2 learners.
3	The Impact of EFL Learners’ Negative Emotional Orientations on (Un)Willingness to Communicate in In-person and Online L2 Learning Contexts	MacIntyre et al.’s (1998) model of WTC; the control–value theory of achievement; the self-determination theory (SDT); the individuals’ psychological need for relatedness, competence, and autonomy	290 university students majoring English language teaching at various universities (males = 102; females = 188).	L2 WTC scale (Lee & Hsieh, 2019); L2 boredom scale (C. Li et al., 2021); L2 anxiety scale (Botes et al., 2022); L2 demotivation scale (Kikuchi, 2015).	SEM; CFA mediation analysis.	EFL learners’ levels of anxiety and demotivation can directly influence in-person and online communication willingness, with L2 demotivation being the strongest negative predictor of face-to-face L2WTC. L2 anxiety was identified as the strongest negative predictor of online L2WTC.
4	Affective variables and willingness to communicate of EFL learners in in-class, out-of-class, and digital contexts	The pyramid model of WTC	261 EFL undergraduate students (males = 84; females = 177).	L2 WTC scale (Lee & Lee, 2020; Peng, 2013); L2 self-confidence scale (Pyun et al., 2014); L2 anxiety scale (Horwitz et al., 1986; Pyun et al., 2014); L2 motivation scale (Gardner, 1985; Gardner et al., 1997; Pyun et al., 2014); Grit scale (A. L. Duckworth & Quinn, 2009).	EFA. Pearson’s Correlation. Three hierarchical (blocked) multiple regression analyses.	Students with higher levels of grit and L2 confidence had higher L2 WTC in all three communicative settings. Lack of L2 anxiety was a significant predictor of students’ L2 WTC in non-digital environments (in-class and out-of-class contexts), but not in the digital setting.
5	Modeling the Contribution of Grit, Enjoyment, and Boredom to Predict English as a Foreign Language Students’ Willingness to Communicate in a Blended Learning Environment	The broaden-and-build theory; Positive Psychology theory; the control–value theory	345 EFL students at several campuses of a public university in Saudi Arabia. (females = 252; males = 93) All grouped at the B1 level.	L2 WTC scale (Y. Wang et al., 2021; Lee & Drajati, 2019a); Grit scale (Teimouri et al., 2022); foreign language enjoyment scale (Botes et al., 2021); foreign language learning boredom scale (Alanazi & Bensalem, 2024; Schwartze et al., 2020).	SEM and CFA via AMOS 26.0; statistical analyses via SPSS 27.0; Pearson correlation analysis.	FLB had the largest relationship to students’ L2 WTC followed by FLE and grit, respectively.
6	Dynamicity of EFL learners’ willingness to communicate in an online class	The Complex Dynamic Systems Theory; MacIntyre et al.’s (1998) model; EFL-based L2 WTC models; the theory of L2 emotional contagion	7 Chinese EFL students.	Participants self-rated their WTC levels while watching a video recording of L2 performance.	The idiodynamic method.	EFL learners’ levels of L2 WTC are highly dynamic as a result of the combined influences of various trait-like (e.g., openness to a new online learning experience) and state-like factors (e.g., technical issues) factors during their participation in an online class.
7	Informal digital learning of English and EFL learners’ willingness to communicate: investigating the mediating role of L2 grit	The theories of digital language learning, affective dimensions, and informal language learning	313 Iran EFL learners	WTC scale (MacIntyre et al., 2001); L2 Grit Scale (Teimouri et al., 2022); Digital Informal Learning Scale (MacIntyre et al., 2001).	SEM with AMOS 26.0 software; CFA;	The correlations between L2 Grit, IDLE, and L2 WTC were significant. The mediating effect of L2 grit on the relationship between IDLE and L2 WTC was statistically significant.
8	Foreign Language Enjoyment as a mediator between Informal Digital Learning of English and willingness to communicate	The broaden-and-build theory; the Positive Psychology theory; the pyramid model of L2 WTC	308 EFL Hong Kong secondary students ages 12–18 (females = 148; males = 160).	L2 WTC scale (Lee, 2022b); IDLE scale (Lee, 2022a); FLE scale (Botes et al., 2021).	CFA using Jamovi 2.3.6	The relationship between receptive IDLE and L2 WTC inside the classroom is partially mediated by teacher appreciation, personal enjoyment, and social enjoyment. The relationship between productive IDLE and L2 WTC inside the classroom is partially mediated by teacher appreciation, personal enjoyment, and social enjoyment.
9	Informal Digital Learning of English and willingness to communicate in a second language: self-efficacy beliefs as a mediator	The social cognitive theory; The pyramid model of WTC	246 Kazakhstani university EFL students (females = 219; males = 27).	L2 WTC scale (Lee & Drajati, 2019a); IDLE scale (Lee & Drajati, 2019a); Self-efficacy beliefs scale (Chacón, 2005).	CFA; SEM.	EFL students who participate in IDLE activities more frequently have a stronger belief in their own ability to perform various tasks in English, which leads to a greater willingness to communicate.
10	Understanding willingness to communicate in L2 between Korean and Taiwanese students	MacIntyre et al.’s (1998) L2 WTC model	143 Korean university students (females = 99; males = 44); 261 Taiwanese university students (females = 177; males = 84).	L2 WTC scale (Ryan, 2009; Yashima, 2009; Peng, 2013; Lee & Lee, 2020).	Exploratory factor analysis (EFA).	The Taiwanese are more willing to communicate in digital settings than the Koreans.
11	Enjoyment and Grit as mediators between informal digital learning English, basic psychology needs, and willingness to communicate among English as a foreign language university students	The pyramid model of L2 WTC; the broaden-and-build theory; the Positive Psychology theory the self-determination theory	1056 Chinese university students learning English as a foreign language (males = 562; females = 494).	L2 WTC scale (Lee & Drajati, 2019b); IDLE scale (Lee et al., 2024b); Foreign Language Enjoyment scale (C. Li et al., 2018); Grit scale (A. Duckworth et al., 2007); Basic Psychological Needs in Second Language scale (Alamer, 2021).	Partial least squares structural equation modeling (PLS-SEM).	Female EFL learners exhibit a greater WTC compared to their male counterparts. IDLE directly and positively predicts WTC in all three communicative contexts. BPNs positively predict WTC in EFL contexts. FLE mediates the relationship between IDLE and WTC within the EFL context. Grit serves as a mediator between IDLE and WTC in the EFL context. FLE serves as a mediator in the relationship between BPNs and WTC in the EFL context. Grit acts as a mediator between BPNs and WTC in the EFL context.
12	Informal digital learning of English and L2 willingness to communicate: roles of emotions, gender, and educational stage	MacIntyre et al.’s (1998) pyramid model	1265 Korean EFL learners (764 secondary and 501 tertiary students; 400 males and 865 females).	L2 WTC scale (Lee et al., 2022; Lee, 2019); IDLE scale (Lee & Drajati, 2019a); L2 enjoyment scale (Dewaele & MacIntyre, 2014); L2 anxiety scale (MacIntyre & Gardner, 1994).	EFA; CFA; SEM with AMOS 26.0 software	L2 enjoyment and L2 anxiety partially mediated the relationship between IDLE and L2 WTC. L2 enjoyment was a stronger mediator than L2 anxiety. IDLE affected females more than males in terms of reducing their L2 anxiety. L2 emotions played a similar mediation role for secondary and tertiary students.
13	A Longitudinal Analysis of Informal Digital Learning of English, Willingness to Communicate and Foreign Language Boredom: A Latent Change Score Mediation Model	Peer contagion; The Complex Dynamic Systems Theory	325 Iranian university students (females = 197; males = 128) The learners’ English proficiency ranged from lower intermediate to upper-intermediate, as evaluated by the Oxford Placement Test, and their ages ranged from 18 to 33.	L2 WTC scale (Lee, 2022b); informal digital learning of English scale (Lee, 2022a).	Longitudinal development of constructs; SEM: latent change score mediation (LCSM) model.	FLB mediates between IDLE and L2 WTC. 3 variables change over time and change more obviously at the early stages compared with later phases of the course.
